# From the Minutes of the Regular Annual Meeting of the West Texas Medical Association

**Published:** 1887-01

**Authors:** Frederick Terrell

**Affiliations:** Secretary


### MINUTES OF THE REGULAR ANNUAL MEETING OF THE WEST TEXAS MEDICAL ASSOCIATION.

*By Frederick Terrell, M. D., Secretary.*

For Daniel's Texas Medical Journal.

SAN ANTONIO, TEXAS, January 1, 1887.

At the regular meeting of the West Texas Medical Association, the following resolutions were unanimously adopted :

"That in the removal from our midst of our esteemed friend and fellow member, Dr. T. J. Tyner, we deplore the loss of a successful practitioner, an eminently skillful operator, and an honorable member of the profession, and we take this opportunity of expressing our high appreciation of his services, in this association, to the cause of legitimate medicine.

*Resolved*, That he carries with him the earnest wishes of this association for his health, prosperity, and happiness.

*Resolved*, That we congratulate the members of the profession in Austin on this valuable accession to their ranks, and the State Institution for the Blind on securing the services of so competent and able a surgeon.

*Resolved*, That the Secretary is hereby instructed to furnish copies of these resolutions to the Travis County Medical Association, and for publication, to DANIEL'S TEXAS MEDICAL JOURNAL, and the Texas Courier Record of Medicine."

